# Layer-by-Layer Self-Assembling Gold Nanorods and Glucose Oxidase onto Carbon Nanotubes Functionalized Sol-Gel Matrix for an Amperometric Glucose Biosensor

**DOI:** 10.3390/nano5031544

**Published:** 2015-09-18

**Authors:** Baoyan Wu, Shihua Hou, Zhiying Miao, Cong Zhang, Yanhong Ji

**Affiliations:** 1 MOE Key Laboratory of Laser Life Science and Institute of Laser Life Science, College of Biophotonics, South China Normal University, Guangzhou 510631, China; E-Mails: wubaoyan@scnu.edu.cn (B.W.); jiyh@scnu.edu.cn (Y.J.); 2 School of Electronic and Information Engineering, South China University of Technology, Guangzhou 510640, China; 3 The key Laboratory of Bioactive Materials Ministry of Education, College of Life Sciences, Nankai University, Tianjin 300071, China; E-Mails: mzy@mail.nankai.edu.cn (Z.M.); zhangcong0672@163.com (C.Z.)

**Keywords:** biosensor, layer-by-layer, sol-gel, gold nanorods (AuNRs), carbon nanotubes

## Abstract

A novel amperometric glucose biosensor was fabricated by layer-by-layer self-assembly of gold nanorods (AuNRs) and glucose oxidase (GOD) onto single-walled carbon nanotubes (SWCNTs)-functionalized three-dimensional sol-gel matrix. A thiolated aqueous silica sol containing SWCNTs was first assembled on the surface of a cleaned Au electrode, and then the alternate self-assembly of AuNRs and GOD were repeated to assemble multilayer films of AuNRs-GOD onto SWCNTs-functionalized silica gel for optimizing the biosensor. Among the resulting glucose biosensors, the four layers of AuNRs-GOD-modified electrode showed the best performance. The sol-SWCNTs-(AuNRs-GOD)_4_/Au biosensor exhibited a good linear range of 0.01–8 mM glucose, high sensitivity of 1.08 μA/mM, and fast amperometric response within 4 s. The good performance of the proposed glucose biosensor could be mainly attributed to the advantages of the three-dimensional sol-gel matrix and stereo self-assembly films, and the natural features of one-dimensional nanostructure SWCNTs and AuNRs. This study may provide a new facile way to fabricate the enzyme-based biosensor with high performance.

## 1. Introduction

The blood glucose level is usually used as a clinical indicator of diabetes mellitus, which is a global health problem with devastating social and economic impact [1]. Amperometric glucose oxidase electrodes have played a leading role in blood sugar testing and point-of-care diagnostics [2]. Immobilizing enzymes efficiently on the electrode surface is one of the most challenging tasks in biosensor fabrication [3]. Both the substrate and the immobilization of enzyme on the substrate is critical because performance is significantly affected by electron transfer and binding strength between the enzyme and electrode [4].

With the advent of nanotechnology, it became clear that the conductive nanomaterials-treated electrode can provide favorable conditions for efficient electron exchange between the electrode substrate and enzyme, and can significantly improve the sensitivity [5,6,7]. One-dimensional (1D) nanostructures such as nanorods, nanowires, and nanotubes can offer direct and fast electron transport to the electron collecting electrode [8,9]. In particular, gold nanorods (AuNRs) have been proved to be versatile and tunable materials compared to other materials including spherical nanoparticles; especially in the detection field due to the shape anisotropy of nanorods [10], AuNRs, the elongated gold nanoparticles, have been used to immobilize biological molecules in order to fabricate different biosensors due to their unique characteristics such as good electron mediation capability, biocompatibility, and the ease of surface modification [11,12,13]. AuNRs-based hybrid nanostructures were found to be a novel biocompatible nanocomposite material used in electrochemical sensors. For example, Li *et al.* fabricated a new glucose electrochemical biosensor by using the composite membrane of multi-walled carbon nanotubes (MWCNTs) and AuNRs to immobilize glucose oxidase onto the electrode surface, in which glucose oxidase could keep its own activities and the degeneration of glucose oxidase did not happened [14]. Recently, the electrochemical indomethacin biosensor was developed using AuNRs and graphene oxide-incorporated carbon nanotube paste-modified glassy carbon electrodes [15]. Meanwhile, Goulart *et al.* reported a nanohybrid platform based on MWCNTs and AuNRs, and showed good electrocatalytic properties of l-cysteine [16]. AuNRs-based nanocomposites can achieve even broader functionalities and applications because of the unique inherent physical properties originating from the one-dimensional shape of nanoparticles [17].

However, these 1D nanostructures have the major disadvantage of having insufficient surface area for enzyme immobilization, and this must be overcome before 1D nanostructures can be applied to the construction of biosensors [8,18]. The coupling of sol-gel and the self-assembly process is an effective method for the preparation of three-dimensional sol-gel network and multilayer films, which can effectively adsorb enzyme, and optimize enzyme loading. One of the quality examples was the fabrication of a horseradish peroxidase amperometric biosensor developed by self-assembling gold nanoparticles to a sol-gel network, which exhibited high sensitivity, good reproducibility, and long-term stability [19]. We previously demonstrated that a favorable bienzyme amperometric acetylcholine biosensor using the self-assembly process in combination with the sol-gel technique achieved the optimum acetylcholinesterase loading [20]. Meanwhile, these nanoparticle ensembles deposited on electrodes can increase the surface area of the electrode by generating a porous surface, and can provide intimate contact with enzymes [21]. Therefore, the three-dimensional sol-gel network and multilayer films used as the immobilization matrix can overcome the shortcomings of 1D nanostructures, and also can develop the advantages of 1D nanostructures.

In this study, we utilized the coupling of sol-gel and the layer-by-layer self-assembly process to fabricate a novel amperometric glucose biosensor based on 1D nanostructures AuNRs and single-walled carbon nanotubes (SWCNTs) for meeting the demand of the effective substrate and the immobilization of enzyme on the substrate. The resulting biosensor should provide new thoughts for the development of the amperometric biosensors based on 1D nanostructures, which can exhibit the benefits of the advantages of 1D hybrid nanostructures, the three-dimensional sol-gel matrix, and stereo self-assembly films.

## 2. Results and Discussion

### 2.1. Preparation and Morphology Characterization

**Figure 1:**
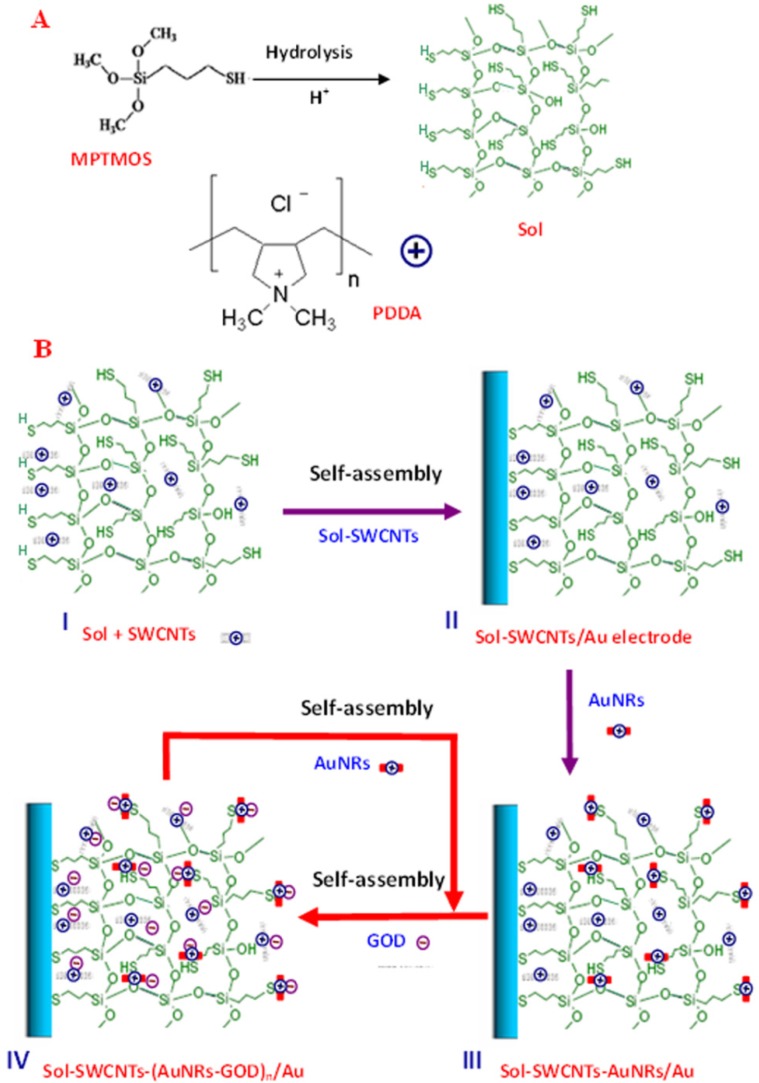
(**A**) Hydrolysis of (3-mercaptopropyl) trimethoxy silane (MPTMOS) and the molecular structure of Poly(diallyldimethylammonium chloride) (PDDA); (**B**) The stepwise fabrication process of the biosensor based on stereo self-assembling gold nanorods (AuNRs) and glucose oxidase (GOD) to single-walled carbon nanotubes (SWCNTs) functionalized three-dimensional sol-gel matrix.

Experiments were carried out with the aim of developing a novel amperometric glucose biosensor based on stereo layer-by-layer self-assembling AuNRs and glucose oxidase (GOD) to SWCNTs-functionalized three-dimensional sol-gel matrix, as shown in Figure 1. Here, a thiolated aqueous silica sol was not only used for immobilization of SWCNTs, but also used to assemble AuNRs via the Au–S bond. Poly(diallyldimethylammonium chloride) (PDDA) on the surface of AuNRs and SWCNTs can provide a friendly interface for the assembly of GOD through electrostatic interaction between positively charged PDDA and negatively charged GOD [22]. Sol containing SWCNTs was firstly assembled on the surface of a cleaned Au electrode, the sol-SWCNTs/Au Electrode. Then the alternate self-assembly of PDDA-AuNRs and GOD was on the sol-SWCNTs/Au electrode surface to obtain the sol-SWCNTs-(AuNRs-GOD)*_n_*/Au electrode. This hydrogel is frequently used in biomedical applications and is an ideal material for enzyme immobilization because of its nontoxic nature and good biocompatibility [2]. Compared to the immobilization of enzymes onto substrate surface, the incorporation of enzymes into the matrix has the potential to increase the enzyme loading as well as to protect the enzyme from the surrounding environment [23].

**Figure 2:**
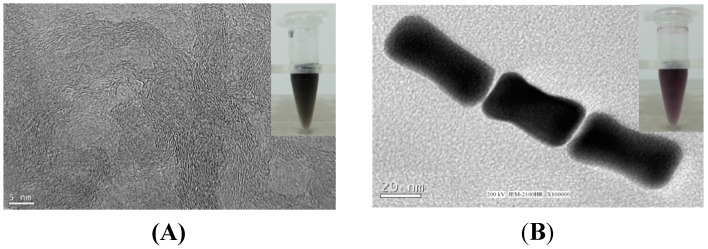
TEM images of (**A**) SWCNTs and (**B**) AuNRs.

Considering the biotoxicity of cetyltrimethylammonium bromide (CTAB) in AuNRs solution [24,25], the positively charged PDDA was used as the specific ligand for functionalized conjugation on AuNRs surfaces to reduce the biotoxicity of AuNRs, and was also used to produce a stable aqueous PDDA-SWCNTs suspension. SWCNTs and AuNRs were solubilized in the aqueous PDDA solution, evidenced by TEM measurement and the homogeneous dark SWCNTs suspension (Figure 2A) and deep purple AuNRs suspension (Figure 2B). The results indicated SWCNTs can exist both individually and in small bundles, indicating a satisfactory dispersibility. AuNRs are rod-like. The average diameter of nanorods is 20 nm, and the average length is 45 nm. It has been demonstrated that small-sized gold nanoparticles can be both diffused into and on the surface of the sol-gel matrix [20]. Therefore SWCNTs and AuNRs used in this work can form a continuous array of SWCNTs and AuNRs in the sol-gel matrix, providing a favorable microenvironment.

### 2.2. UV-Vis Analysis

**Figure 3:**
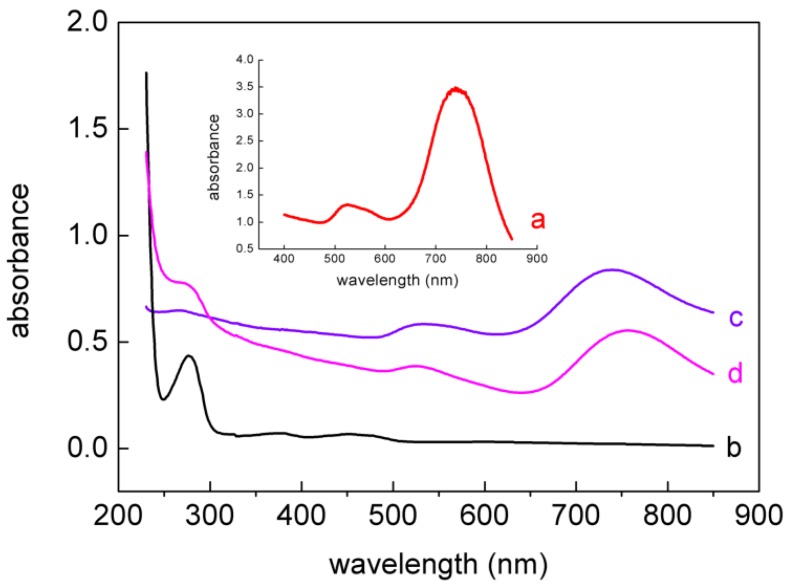
UV-Vis absorption spectra of (**a**) bare AuNRs; (**b**) pure GOD; (**c**) PDDA-AuNRs; and (**d**) PDDA-AuNRs-GOD.

UV-visible absorption spectroscopy is a useful tool to monitor the AuNRs and GOD. Figure 3 shows the absorption spectra of (a) bare AuNRs, (b) pure GOD, (c) PDDA-AuNRs, and (d) PDDA-AuNRs-GOD. The characteristic absorption spectrum of bare AuNRs was observed at a strong long-wavelength band around 641 nm due to the longitudinal oscillation of electrons and a weak short-wavelength band around 516 nm due to the transverse electronic oscillation. Pure GOD had a sharp absorption peak at 277 nm along with a pair of peaks at 373 nm and 471 nm, which were consistent with previous reports [9,26]. As seen from curve c and d, the characteristic absorption peaks of AuNRs and GOD remained in PDDA-AuNRs-GOD, indicating that GOD had been adsorbed on the surface of PDDA-AuNRs.

### 2.3. Cyclic Voltammetric Characterization

**Figure 4:**
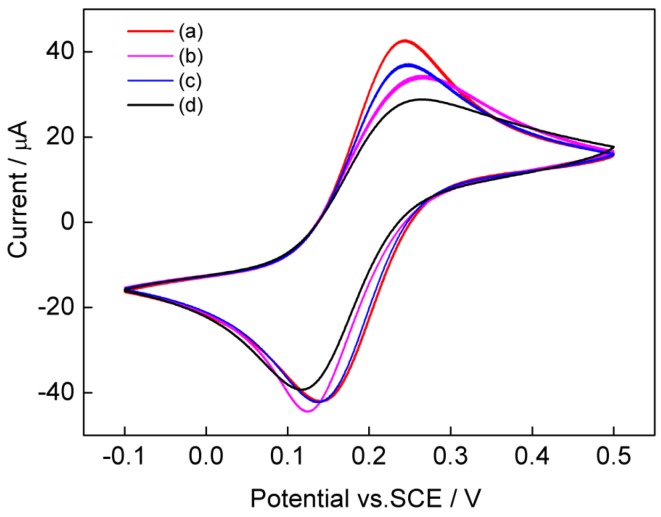
Cyclic voltammograms of the (**a**) bare Au electrode; (**b**) sol-SWCNTs/Au; (**c**) sol-SWCNTs-AuNRs/Au; and (**d**) sol-SWCNTs-AuNRs-GOD/Au in 0.1 M KCl containing 5 mM [Fe (CN)_6_]^3−/4−^ (1:1) with a scan rate of 50 mV/s.

Cyclic voltammetry of [Fe(CN)_6_]^3−/4−^ is a valuable tool for testing the changes of electrode behavior after each assembly step of the modified electrode. Figure 4 shows the cyclic voltammograms of the (a) bare electrode, (b) sol-SWCNTs/Au, (c) sol-SWCNTs-AuNRs/Au, and (d) sol-SWCNTs-AuNRs-GOD/Au in 0.1 M KCl containing 5 mM [Fe (CN)_6_]^3−/4−^ (1:1) with a scan rate of 50 mV/s. A pair of redox peaks corresponding to the redox reaction of [Fe(CN)_6_]^3−/4−^ was observed at the bare Au electrode (curve a). When the electrode was modified with sol-SWCNTs, the peak currents decreased, and the peak-to-peak separation increased (curve b). This may be due to the insulation properties of the sol membrane [19]. Compare to the sol-SWCNTs electrode, the peak currents of the sol-SWCNTs-AuNRs electrode increased (curve c), indicating the enhancing effect of AuNRs on the electric conductivity of the enzyme electrode. When GOD was further assembled on the sol-SWCNTs-AuNRs (curve d), the peak currents decreased, mainly due to the GOD-reduced electron transfer between anionic [Fe(CN)_6_]^3−/4−^ and the electrode. Taken together, the absorbance spectra and cyclic voltammetry data showed that AuNRs and GOD were successfully assembled into the SWCNTs-functionalized three-dimensional sol-gel matrix.

### 2.4. Effect of AuNRs-GOD Multilayers

**Figure 5:**
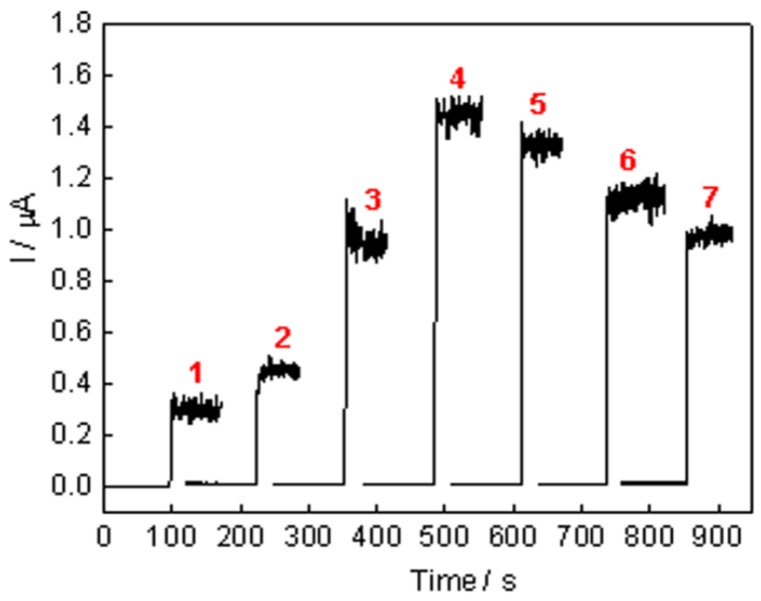
Amperometric responses of different layers of AuNRs-GOD bilayer films-modified Au electrode in 1 mM glucose (number of AuNRs-GOD indicated on graph).

To optimize the number of AuNRs-GOD bilayers, the amperometric responses of the (AuNRs-GOD)*_n_*-modified electrodes were studied systematically as a function of the number of bilayers at the applied potential of 0.6 V *vs.* saturated calomel electrode (SCE) under gently magnetic stirring, as shown in Figure 5. Curves 1–7 displayed the amperometric response of the 1–7 layers of the AuNRs-GOD-modified Au electrode to 1 mM glucose in phosphate-buffered saline (PBS, pH 7.0) buffer, respectively. The amperometric glucose biosensor originates from the oxidation current of H_2_O_2_, which generates during the course of the GOD-catalyzed oxidation of glucose in the presence of dissolved oxygen [27]. All the biosensors showed rapid responses upon the addition of glucose, achieving a steady-state current in less than 4 s. The fast response might be due to the excellent electron transfer ability of carbon nanotubes and gold nanoparticles [28]. The amperometric response of the resulting glucose biosensor to glucose increased with the number of AuNRs-GOD from one to four layers, which may be attributable to the increased loading of GOD. The highest response was observed for the sol-SWCNTs-(AuNRs-GOD)_4_/Au electrode, and then gradually decreased upon further increasing the number of bilayers, which may be because the substrates of the enzymes and the reaction products cannot pass smoothly through the thicker films [29].

### 2.5. Chronoamperometric Response

**Figure 6:**
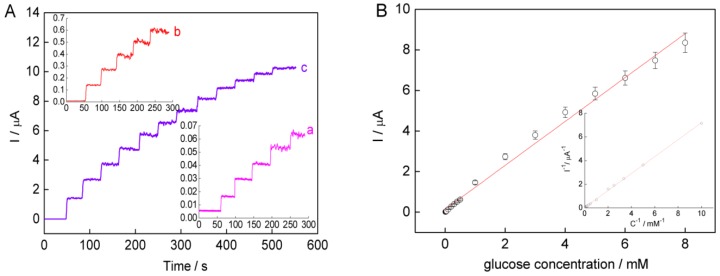
(**A**) Current-time curves obtained at the sol-SWCNTs-(AuNRs-GOD)_4_/Au electrode for successive additions of 0.01 mM (curve a), 0.1 mM (curve b), and 1 mM (curve c) glucose in PBS at 0.35 V *vs*. saturated calomel electrode; (**B**) The calibration curve of the enzyme electrode as a function of glucose concentrations. Inset shows Lineweaver-Burk plot for the determination of $K_{\text{m}}^{\text{app}}$. Error bar = standard deviation (*n* = 5).

Figure 6A showed the amperometric responses obtained for successive additions of 0.01 mM glucose (curve a), 0.1 mM glucose (curve b), and 1 mM glucose (curve c) at the sol-SWCNTs-(AuNRs-GOD)4/Au electrode in PBS at the applied potential of 0.35 V *vs.* SCE under gentle magnetic stirring. Well-defined amperometric step responses were observed for each glucose injection. The linear dependence of current with glucose concentration is given in Figure 6B. The linear range scans the glucose concentration from 0.01 mM to 8 mM with a sensitivity of 1.08 μA/mM (*n* = 5, *R*^2^ = 0.9923). The detection limit is 4.2 μM at the signal-to-noise ratio of 3. The response current increases linearly along with the increase of glucose concentration and gradually reached a saturation value at high glucose concentration. This is the characteristic of typical Michaelis-Menten kinetics. The apparent Michaelis-Menten constant ($K_{\text{m}}^{\text{app}}$), a reflection of the enzymatic affinity, is calculated to be 15.4 mM according to the Lineweaver-Burk equation (the inset of Figure 6B). The value of $K_{\text{m}}^{\text{app}}$ agrees well with the reported value of 13.9 mM for the carbon nanotubes/polyacrylonitrile/GOD biosensor [30], and is lower than that of 19.2 mM for GOD on poly(methyl methacrylate)-bovine serum albumin core-shell nanoparticles [31], 23 mM for GOD encapsulated in polyvinyl alcohol silica hybrid films [2], 33 mM for GOD entrapped in a hydrogel matrix [32], and 27 mM for GOD itself in solution [33], which indicates that sol-SWNTs-AuNRs-immobilized GOD still possesses high bioactivity.

### 2.6. Selectivity, Reproducibility, and Stability

Three kinds of possible interfering substances, 0.5 mM uric acid, 0.1 mM ascorbic acid, and 0.1 mM acetaminophen, were used for measurement in our experiments, and no noticeable interfering current was detected through measuring the amperometric response to 5 mM glucose. Relative standard deviation of the current response to 5 mM glucose was 7% for six successive measurements, indicating an acceptable reproducibility. In order to evaluate the stability, the amperometric response of the sol-SWCNTs-(AuNRs-GOD)_4_/Au electrode to 5 mM glucose was measured every day. It still remained with 90% of its initial current response after one month. The good performance of the biosensor could be mainly attributed to the advantages of the three-dimensional sol-gel matrix and stereo self-assembly films, and the natural features of one-dimensional nanostructures AuNRs and SWCNTs, which can provide a favorable microenvironment for enzyme immobilization and electron transfer.

## 3. Experimental Section

### 3.1. Reagents

Glucose oxidase from Aspergillus niger (GOD, EC1.1.3.4, 202 units/mg protein) and (3-mercaptopropyl) trimethoxy silane (MPTMOS) were purchased from sigma-Aldrich. SWCNTs (diameter 1–2 nm and length 0.5–2 μm) were obtained from Chengdu Institute of Organic Chemistry (Chengdu, China). HAuCl_4_·4H_2_O, AgNO_3_, NaBH_4_, l-ascorbic acid, and cetyltrimethylammonium bromide (CTAB) were purchased from Sinopharm Chemical Reagent Co., Ltd. (Shanghai, China). All the reagents were of analytical grade and used without further purification. pH 7.0 PBS was employed as supporting electrolyte. All experiments were performed at room temperature, approximately 25 °C.

### 3.2. Apparatus and Measurements

Electrochemical measurements were carried out with CHI800C electrochemical analyzer (Chen Hua Instruments, Shanghai, China). The experiments were performed with a conventional three-electrode system in an electrochemical cell filled with 20 mL of PBS at room temperature. The working electrode was the enzyme-modified Au electrode. A saturated calomel electrode (SCE) and platinum wire electrode were used as reference electrode and auxiliary electrode, respectively. Absorbance measurements were carried out on a UV/VIS spectrometer (Lambda 35, Perkin-Elmer, Norwalk, CT, USA). Transmission electron microscopy (TEM) measurement was carried out on a JEM-2100HR TEM at 200 kV.

### 3.3. Preparation of the Glucose Biosensor

#### 3.3.1. Preparation of PDDA-AuNRs and Sol-SWCNTs

AuNRs were prepared according to the silver ion-assisted seed-mediated method using CTAB as template [34]. The synthesis of the PDDA-AuNRs was as follows: first, the as-prepared AuNRs solution were purified to remove excess free surfactant CTAB by centrifugation at 5000 rpm for 10 min and further washed with water. Second, the purified AuNRs were dispersed in PDDA solution, and kept overnight under stirring. Third, to remove the excess of free PDDA, PDDA-AuNRs suspension was centrifuged twice at 5000 rpm for 10 min, and the precipitate was re-dispersed in PBS. The thiolated aqueous silica sol was prepared according to the literature [19,35]. Sol-SWCNTs were prepared in multistep process as previously reported [20]. In brief, SWCNTs were sonicated in PDDA solution, then centrifuged at 10,000 rpm for 10 min, and the supernatant containing PDDA-SWCNTs was collected. PDDA-SWCNTs were added into the thiolated aqueous silica sol solution, and sonicated for 10 min, yielding a homogeneous black sol-SWCNTs suspension.

#### 3.3.2. Configuration of the Glucose Biosensor

The bare Au electrode was first immersed in the sol-SWCNTs suspension for 30 min, and thoroughly rinsed with PBS, which was applied at the end of each assembly deposition for dissociating the weak adsorption. Afterward, the sol-SWCNTs-modified Au electrode was immersed in PDDA-AuNRs suspension for 5 h, and then transferred to GOD solution for 30 min. The AuNRs-GOD-treated electrode was expressed as sol-SWCNTs-AuNRs-GOD/Au electrode. Finally, the sol-SWCNTs-AuNRs-GOD/Au electrode was alternately immersed in PDDA-AuNRs and GOD solution until desired layer number of AuNRs-GOD was achieved, and the as-prepared electrode was denoted as sol-SWCNTs-(AuNRs-GOD)*_n_*/Au electrode.

## 4. Conclusions

This paper presents a novel amperometric glucose biosensor based on stereo self-assembly multilayers of AuNRs-GOD on the three-dimensional sol-SWCNTs matrix-modified Au electrode surface. Among the resulting biosensors, the biosensor based on four layers of AuNRs-GOD bilayer showed the best performance. The resulting sol-SWCNTs-(AuNRs-GOD)_4_/Au electrode has a fast and sensitive response and a wide linear detection range to glucose with good stability and selectivity. The three-dimensional sol-SWCNTs matrix and stereo self-assembly films of AuNRs-GOD can provide a promising material for the biosensor designs and other biological applications. The general idea and preparation method presented in this study may offer a new facile way to fabricate the enzyme-based biosensor with high performance.

## References

[1] Guo X.S., Liang B., Jian J.M., Zhang Y.L., Ye X.S. (2014). Glucose biosensor based on a platinum electrode modified with rhodium nanoparticles and with glucose oxidase immobilized on gold nanoparticles. Microchim. Acta.

[2] Lad U., Kale G.M., Bryaskova R. (2013). Glucose oxidase encapsulated polyvinyl alcohol silica hybrid films for an electrochemical glucose sensing electrode. Anal. Chem..

[3] Unnikrishnan B., Palanisamy S., Chen S.M. (2013). A simple electrochemical approach to fabricate glucose a biosensor based on graphene–glucose oxidase biocomposite. Biosens. Bioelectron..

[4] Jung J., Lim S. (2013). ZnO nanowire-based glucose biosensors with different coupling agents. Appl. Surf. Sci..

[5] Guo M.Q., Fang H.D., Wang R., Yang Z.Q., Xu X.H. (2011). Electrodeposition of chitosan-glucose oxidase biocomposite onto Pt-Pb nanoparticles modified stainless steel needle electrode for amperometric glucose biosensor. J. Mater. Sci. Mater. Med..

[6] Szot K., Jönsson-Niedziolka M., Rozniecka E., Marken F., Opallo M. (2013). Direct electrochemistry of adsorbed proteins and bioelectrocatalysis at film electrode prepared from oppositely charged carbon nanoparticles. Electrochim. Acta.

[7] Jin L.Y., Gao X., Chen H., Wang L.S., Wu Q., Chen Z.C., Lin X.F. (2013). Efficient immobilization of glucose oxidase on nanocomposite via layer-by-layer self-assembly to construct bionanomultilayer films. J. Electrochem. Soc..

[8] Poudel P., Qiao Q.Q. (2012). One dimensional nanostructure/nanoparticle composites as photoanodes for dye-sensitized solar cells. Nanoscale.

[9] Ren X.L., Chen D., Meng X.W., Tang F.Q., Du A.M., Zhang L. (2009). Amperometric glucose biosensor based on a gold nanorods/cellulose acetate composite film as immobilization matrix. Colloid Surface. B..

[10] Wang Y.S., Zhang D.H., Liu W., Zhang X., Yu S.X., Liu T., Zhang W.T., Zhu W.X., Wang J.H. (2014). Facile colorimetric method for simple and rapid detection of endotoxin based on counterion-mediated gold nanorods aggregation. Biosens. Bioelectron..

[11] Wang C.G., Ma Z.F., Wang T.T., Su Z.M. (2006). Synthesis, assembly, and biofunctionalization of silica-coated gold nanorods for colorimetric biosensing. Adv. Funct. Mater..

[12] Komathi S., Gopalan A.I., Kim S.K., Anand G.S., Lee K.P. (2013). Fabrication of horseradish peroxidase immobilized poly (*N*-[3-(trimethoxy silyl)propyl]aniline) gold nanorods film modified electrode and electrochemical hydrogen peroxide sensing. Electrochim. Acta.

[13] Vigderman L., Khanal B.P., Zubarev E.R. (2012). Functional gold nanorods: Synthesis, self-assembly, and sensing applications. Adv. Mater..

[14] Li Y.C., Wang F., Huang F.Y., Li Y.J., Feng S.Q. (2012). Direct electrochemistry of glucose oxidase and its biosensing to glucose based on the Chit-MWCNTs-AuNRs modified gold electrode. J. Electroanal. Chem..

[15] Arvand M., Gholizadeh T.M. (2013). Gold nanorods-graphene oxide nanocomposite incorporated carbon nanotube paste modified glassy carbon electrode for voltammetric determination of indomethacin. Sensors Actuators B.

[16] SantosSilva F.A., AngelodaSilva M.G., Lima P.R., Meneghetti M.R., Kubota L.T., Goulart M.F. (2013). A very low potential electrochemical detection of l-cysteine based on a glassy carbon electrode modified with multi-walled carbon nanotubes/gold nanorods. Biosens. Bioelectron..

[17] Bao Y., Vigderman L., Zubarev E.R., Jiang C.Y. (2012). Robust multilayer thin films containing cationic thiol-functionalized gold nanorods for tunable plasmonic properties. Langmuir.

[18] Li W.K., Zhang P., Dai M., He J., Babu T., Xu Y.L., Deng R.H., Liang R.J., Lu M.H., Nie Z.H. (2013). Ordering of gold nanorods in confined spaces by directed assembly. Macromolecules.

[19] Jia J., Wang B., Wu A., Cheng G., Li Z., Dong S. (2002). A method to construct a third-generation horseradish peroxidase biosensor: Self-assembling gold nanoparticles to three-dimensional sol-gel network. Anal. Chem..

[20] Hou S.H., Ou Z.M., Chen Q., Wu B.Y. (2012). Amperometric acetylcholine biosensor based on self-assembly of gold nanoparticles and acetylcholine esterase on the sol-gel/multi-walled carbon nanotubes/choline oxidase composite-modified platinum electrode. Biosens. Bioelectron..

[21] Nie Z.H., Petukhova A., Kumacheva E. (2010). Properties and emerging applications of self-assembled structures made from inorganic nanoparticles. Nat. Nanotechnol..

[22] Wu B.Y., Hou S.H., Yin F., Li J., Zhao Z.X., Huang J.D., Chen Q. (2007). Amperometric glucose biosensor based on layer-by-layer assembly of multilayer films composed of chitosan, gold nanoparticles and glucose oxidase modefied Pt electrode. Biosens. Bioelectron..

[23] Qu F.L., Zhang Y., Rasooly A., Yang M.H. (2014). Electrochemical biosensing platform using hydrogel prepared from ferrocence modified amino acid as highly efficient immobilization. Anal. Chem..

[24] Zhang J.J., Liu Y.G., Jiang L.P., Zhu J.J. (2008). Synthesis, characterizations of silica-coated gold nanorods and its applications in electroanalysis of hemoglobin. Electrochem. Commun..

[25] Cai H.H., Lin D.W., Wang J.H., Yang P.H., Cai J.Y. (2014). Controlled side-by-side assembly of gold nanorods: A strategy for lead detection. Sensors Actuators B.

[26] Zhou K.F., Zhu Y.H., Yang X.L., Li C.Z. (2010). Electrocatalytic oxidation of glucose by the glucose oxidase immobilized in graphene-Au-Nafion biocomposite. Electroanalysis.

[27] Hasan K.U., Asif M.H., Hassan M.U., Sandberg M.O., Nur O., Willander M., Fagerholm S., Strålfors P. (2015). A miniature graphene-based biosensor for intracellular glucose measurements. Electrochim. Acta.

[28] Wu B.Y., Hou S.H., Yin F., Zhao Z.X., Wang Y.Y., Wang X.S., Chen Q. (2007). Amperometric glucose biosensor based on multilayer films via layer-by-layer self-assembly of multi-wall carbon nanotubes, gold nanoparticles and glucose oxidase on the Pt electrode. Biosens. Bioelectron..

[29] Wang Y.Y., Wang X.S., Wu B.Y., Zhao Z.X., Yin F., Li S., Qin X., Chen Q. (2008). Dispersion of single-walled carbon nanotubes in poly(diallyldimethylammonium chloride) for preparation of a glucose biosensor. Sensors Actuators B.

[30] Nenkova R., Ivanova D., Vladimirova J., Godjevargova T. (2010). New amperometric glucose biosensor based on cross-linking of glucose oxidase on silica gel/multiwalled carbon nanotubes/polyacrylonitrile nanocomposite film. Sensors Actuators B.

[31] He C.X., Liu J.H., Zhang Q.L., Wu C. (2012). A novel stable amperometric glucose biosensor based on the adsorption of glucose oxidase on poly(methyl methacrylate)-bovine serum albumin core-shell nanoparticles. Sensors Actuators B.

[32] Kotanen C.N., Tlili C., Guiseppi-Elie A. (2013). Amperometric glucose biosensor based on electroconductive hydrogels. Talanta.

[33] Rogers M.J., Brandt K.G. (1971). Interaction of d-glucal with *Aspergillus niger* glucoseoxidase. Biochemistry.

[34] Nikoobakht B., El-Sayed M.A. (2003). Preparation and growth mechanism of gold nanorods using seed-mediated growth method. Chem. Mater..

[35] Wang B., Li B., Deng Q., Dong S.J. (1998). Amperometric biosensor based on sol-gel organic-inorganic hybrid material. Anal. Chem..

